# Differential remodeling of the electron transport chain is required to support TLR3 and TLR4 signaling and cytokine production in macrophages

**DOI:** 10.1038/s41598-019-55295-4

**Published:** 2019-12-11

**Authors:** Duale Ahmed, David Roy, Allison Jaworski, Alexander Edwards, Alfonso Abizaid, Ashok Kumar, Ashkan Golshani, Edana Cassol

**Affiliations:** 1 Department of Biology, Carleton University, Ottawa, Ontario Canada; 2 Department of Health Sciences, Carleton University, Ottawa, Ontario Canada; 3 Department of Neuroscience, Carleton University, Ottawa, Ontario Canada; 4 Department of Pathology and Laboratory Medicine, Faculty of Medicine, University of Ottawa, Ottawa, Ontario Canada; 5 The Children’s Hospital of Eastern Ontario Research Institute, Ottawa, Ontario Canada

**Keywords:** Toll-like receptors, Monocytes and macrophages

## Abstract

Increasing evidence suggests that mitochondria play a critical role in driving innate immune responses against bacteria and viruses. However, it is unclear if differential reprogramming of mitochondrial function contributes to the fine tuning of pathogen specific immune responses. Here, we found that TLR3 and TLR4 engagement on murine bone marrow derived macrophages was associated with differential remodeling of electron transport chain complex expression. This remodeling was associated with differential accumulation of mitochondrial and cytosolic ROS, which were required to support ligand specific inflammatory and antiviral cytokine production. We also found that the magnitude of TLR3, but not TLR4, responses were modulated by glucose availability. Under conditions of low glucose, TLR3 engagement was associated with increased ETC complex III expression, increased mitochondrial and cytosolic ROS and increased inflammatory and antiviral cytokine production. This amplification was selectively reversed by targeting superoxide production from the outer Q-binding site of the ETC complex III. These results suggest that ligand specific modulation of the ETC may act as a rheostat that fine tunes innate immune responses *via* mitochondrial ROS production. Modulation of these processes may represent a novel mechanism to modulate the nature as well as the magnitude of antiviral vs. inflammatory immune responses.

## Introduction

The innate immune system, including tissue macrophages, represent the first line of defence against invading microbial pathogens. Early recognition depends on a variety of pattern recognition receptors (PRRs), which detect evolutionarily conserved structures termed pathogen associated molecular patterns (PAMPs)^1,2^. Key players in this process are Toll-like receptors (TLR), which are capable of detecting a range of PAMPs from viruses and bacteria^1–5^. Among the best characterized are TLR3 and TLR4. TLR3 recognizes double stranded RNA (dsRNA), a common PAMP associated with viral infections^6^. TLR4 primarily recognizes lipopolysaccharide (LPS), the core component of the outer membrane of Gram-negative bacteria^7^. Both TLR3 and TLR4 differentially and dynamically modulate nuclear factor-κB (NF-κB) and interferon regulatory factory 3 (IRF3) signaling following receptor engagement. While TLR3 activates NF-κB and IRF3 signaling *via* TIR-domain-containing adapter-inducing interferon-β protein (TRIF), TLR4 signals through both Myeloid differentiation primary response 88 (MyD88) and TRIF^1^. Differential activation of these signaling pathways plays a critical role in fine tuning pathogen specific antiviral and antibacterial responses^1^.

Cellular metabolism has emerged as a key regulator of macrophage function. Metabolic reprogramming is required to meet the bioenergetic and biosynthetic demands of the cell and to drive effector functions^8–10^. Alterations in metabolites and other bioactive metabolic products have also been shown to activate and regulate gene expression, signal transduction and epigenetic profiles^9,11–14^. Among the best characterized examples of metabolic reprogramming occurs following LPS stimulation^9,14–18^. Almost immediately after TLR4 engagement, macrophages downregulate oxidative phosphorylation (OXPHOS) activity and dramatically increase glycolysis to support rapid ATP production^14,15,17–19^. This repurposing of mitochondrial function also increases reactive oxygen species (ROS) levels, which drives inflammatory cytokine production^9,15,17^. ROS production is driven by the combined effects of increased mitochondrial membrane potential (MMP) and the oxidation of succinate by complex II of the electron transport chain (ETC)^15^ suggesting flux through the ETC may play a central role in this process. However, it is still unclear if the dynamic modulation of ETC complexes and increased ROS production contributes to signaling events following engagement of other TLRs and if differential reprogramming of these processes contributes to ligand specific immune responses.

Mitochondria are known to play an important role in innate immune responses against RNA viruses^20–23^. Recognition of cytosolic viral RNA by retinoic acid-inducible gene I (RIG-I)-like receptors (RLR) and their downstream processes have been shown to require the participation of mitochondrial antiviral signaling (MAVS), a mitochondrial outer membrane adaptor protein^20,24,25^. MAVS acts as a scaffold and recruits effector molecules to the mitochondrial outer membrane leading to the activation of NF-κB and IRF3^20^. More recent studies have shown that mitochondria also contribute to antiviral signaling *via* MAVS independent mechanisms. Tal *et al*. found that mitochondrial ROS (mtROS) potentiates RLR signaling. This signaling is regulated by autophagy and clearance of dysfunctional mitochondria^26^. Alternatively, Yoshizumi *et al*. found that RLR mediated antiviral responses are dependent on OXPHOS activity. This dependence is regulated by the mitochondrial fusion protein optic atrophy 1 (OPA1)^23^. These findings suggest that other aspects of mitochondrial function, independent of MAVS mediated scaffolding, may play a central role in facilitating antiviral responses.

While both TLR3 and RLR recognize and respond to virally derived RNAs, they signal through distinct transduction pathways to trigger antiviral immune responses^27,28^. To date, the role of the mitochondria in driving TLR3 mediated responses in macrophages remains poorly understood. Previous studies have shown that engagement of TLR3 on hepatocytes and dendritic cells (DCs) causes a shift from OXPHOS towards aerobic glycolysis for energy production^29–32^. In DCs, this shift is driven predominately by the *de novo* production of type I interferons (IFN) and therefore is required to rapidly meet the increased energy demands of these activated cells^30–32^. Macrophages stimulated with high concentrations (10 μg/mL) of the synthetic TLR3 agonist polyinosinic-polycytidylic acid (Poly(I:C) or PIC) have been shown to downregulate Complex I-associated ATP production under standard culture conditions^33^. However, the functional consequences of this ETC reprogramming has yet to be elucidated.

In the current study, we used murine bone marrow derived macrophages (BMM) to evaluate how reprogramming of mitochondrial function contributes to TLR3 and TLR4 signaling and cytokine production and how glucose availability affected these responses. We found that modulation of flux through the ETC and associated ROS production plays a critical role in cytokine production following TLR engagement. This reprogramming is ligand specific and may have differential effects on the expression of individual cytokines (e.g. accumulation of mitochondrial vs. cytosolic ROS). Further, low glucose conditions resulted in differential reprogramming of mitochondrial function following TLR3 engagement. This reprogramming upregulated complex III expression and associated mitochondrial ROS production, which amplified inflammatory and antiviral signaling and cytokine production. Collectively, these findings suggest that the ETC may act as a selective rheostat of innate immune responses that differentially regulates ligand specific responses based on nutrient availability.

## Results

### Differential production of pro-inflammatory and antiviral cytokines in PIC- and LPS-stimulated BMM

**Figure 1:**
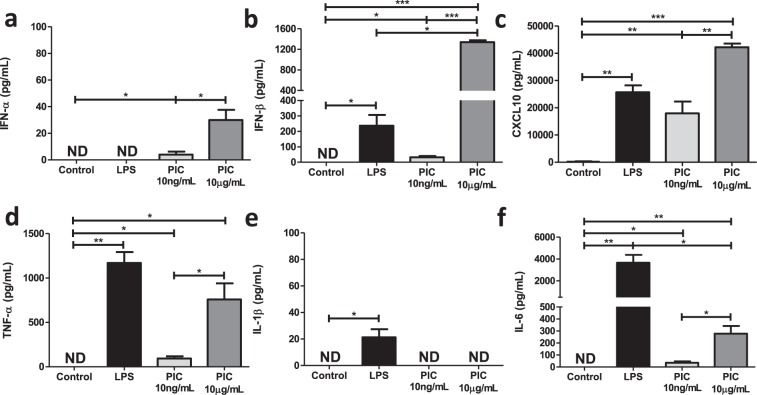
High, but not low, concentrations of Poly(I:C) are associated with pro-inflammatory cytokine production. BMMs were treated with either 100 ng/mL lipopolysaccharide (LPS), 10 ng/mL or 10 μg/mL Poly(I:C) (PIC) for 18 hours. Supernatant was collected and assessed for antiviral (IFN-α, IFN-β, CXCL10) **(a–c)** and pro-inflammatory (TNF-α, IL-1β, IL-6) (**d**–**f**) cytokine expression. Data represents mean ± SEM of four individual mice (*p < 0.05, **p < 0.01, and ***p < 0.001).

Despite activating the same transcription factors (e.g. NF-κB and IRF3), signaling through TLR3 and TLR4 are associated with distinct inflammatory and antiviral cytokine profiles. To evaluate these differences in our model system, we stimulated BMM with PIC (10 ng/ml and 10 μg/ml) or LPS (100 ng/ml) for 18 hours and assessed inflammatory (TNF-α, IL-1β, and IL-6) and antiviral (IFN-α, IFN-β and CXCL10) cytokine production in culture supernatants. PIC concentrations were selected to emulate responses in early (low levels of virus) and late stages of infection (high levels of virus), where Lin *et al*. found that only high (≥10 μg/ml) concentrations of PIC can induce robust inflammatory cytokine production^34^. The LPS concentration was selected based on its ability to repurpose mitochondrial function to support ROS production^15^. As previously reported^15^, LPS induced a strong inflammatory response, produced intermediate levels of IFN-β and CXCL10 and no IFN-α (Fig. 1). Alternatively, stimulation with low concentrations of PIC induced low levels of antiviral and inflammatory production. Increasing the PIC concentration (10 μg/ml) significantly increased both inflammatory and antiviral cytokine production (Fig. 1).

### BMM stimulated with low versus high concentrations of PIC differ in their ability to ramp up glycolytic activity under stress

**Figure 2:**
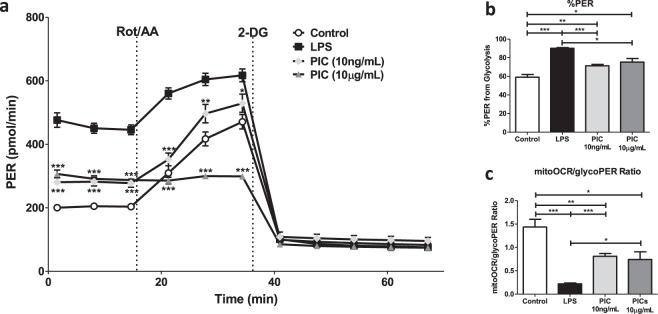
Macrophages activated using higher concentrations of Poly(I:C) are functioning near their maximum glycolytic capacity. BMMs were seeded onto Seahorse XFp miniplates and treated with 100 ng/mL LPS, 10 ng/mL or 10 μg/mL PIC for 18 hours. Glycolytic activity, indicated by the proton efflux rate (PER) was measured using sequential injections of rotenone plus antimycin A (Rot/AA) and 2-deoxyglucose (2-DG) (**a**), determining the %PER dependent on glycolysis **(b)** and the ratio of mitochondrial oxygen consumption rate (mitoOCR) to glycolytic PER **(c)**. Data represents mean ± SEM of four individual mice. The levels of significance shown in (**a**) represent pairwise comparisons against LPS-treated macrophages (*p < 0.05, **p < 0.01, and ***p < 0.001).

Next, we evaluated the differential effects of LPS and PIC stimulation on cellular metabolism using the Seahorse extracellular efflux analyser. As described above, BMM were stimulated with LPS (100 ng/ml), low (10 ng/ml) or high (10 μg/ml) concentrations of PIC for 18 hours. Changes in proton efflux rate (PER) at baseline and in response to rotenone + antimycin (ROT/AA) and 2-deoxyglucose (2-DG) injections were used to evaluate changes in glycolytic parameters. As previously described^9,35^, LPS stimulation increased PER levels at baseline, increased the %PER derived from glycolysis and decreased the mitoOCR/glycoPER ratio suggesting a strong shift away from OXPHOS activity towards aerobic glycolysis (Fig. 2). Stimulation with both concentrations of PIC also increased PER levels at baseline and the %PER derived from glycolysis, but this increase was significantly lower than that observed following LPS-stimulation (P < 0.001). Further, the reduction in the mitoOCR/glycoPER ratio was less pronounced suggesting that PIC-stimulated cells maintain higher levels of OXPHOS activity (Fig. 2c). Despite similar basal PER levels, low and high concentrations of PIC differentially affected the ability of BMM to ramp up glycolysis following stress with Rot/AA. While cells stimulated with lower concentrations maintained their ability to increase glycolytic activity following exposure to Rot/AA, cells stimulated with higher concentrations were unable to do so, suggesting they may be functioning at their maximum glycolytic capacity (Fig. 2a).

### Maintenance of OXPHOS activity is an important feature of PIC but not LPS stimulation

**Figure 3:**
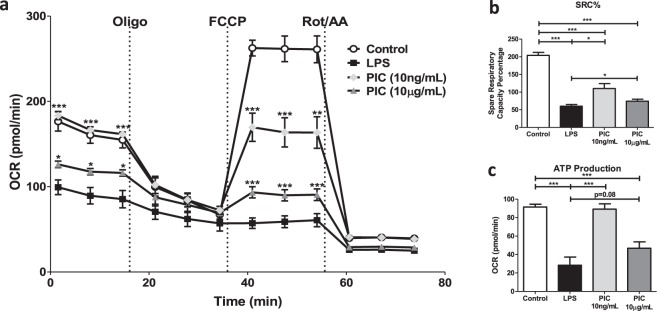
Poly(I:C) stimulation is linked to low sustained levels of oxidative phosphorylation (OXPHOS). Macrophages were plated onto Seahorse XFp miniplates and subsequently stimulated with 100 ng/mL LPS, 10 μg/mL or 10 ng/mL PIC for 18 hours. OXPHOS function was assessed *via* successive Oligomycin (Oligo), Carbonyl cyanide-*p*-trifluoromethoxyphenylhydrazone (FCCP), and Rot/AA injections **(a)**, quantifying the spare respiratory capacity percentage (SRC%) **(b)** and ATP production **(c)**. Data represents mean ± SEM of four individual mice. The levels of significance shown in (**a**) is based on pairwise comparisons to LPS-treated macrophages (*p < 0.05, **p < 0.01, and ***p < 0.001).

To evaluate specific changes in OXPHOS activity, we used the Cell Mito Stress Test kit from Agilent. Features of OXPHOS activity were calculated based on changes in oxygen consumption rate (OCR) in response to successive injections of oligomycin (Oligo), carbonyl cyanide 4-(trifluoromethoxy)phenylhydrazone (FCCP) and ROT/AA. Consistent with the literature^9,19,36^, LPS stimulation dramatically reduced basal respiration, reduced mitochondrial ATP production and reduced the ability of cells to increase oxygen consumption (e.g. spare respiratory capacity [SRC]) following FCCP treatment (Fig. 3). High concentrations of PIC also reduced levels of basal respiration, ATP production and SRC compared to untreated cells but this impairment was less severe than that observed for LPS (basal respiration p < 0.05, SRC p < 0.01, ATP production P = 0.08). Interestingly, low concentrations of PIC did not alter basal respiration or ATP production but significantly reduced SRC suggesting these cells may have a reduced capacity to deal with stress^37^. Given these differences in glycolysis and oxygen consumption, the remaining experiments were performed using low concentration PIC (10 ng/ml).

### Reduced glucose availability is associated with increased inflammatory and antiviral cytokine production in PIC- but not LPS stimulated BMM

**Figure 4:**
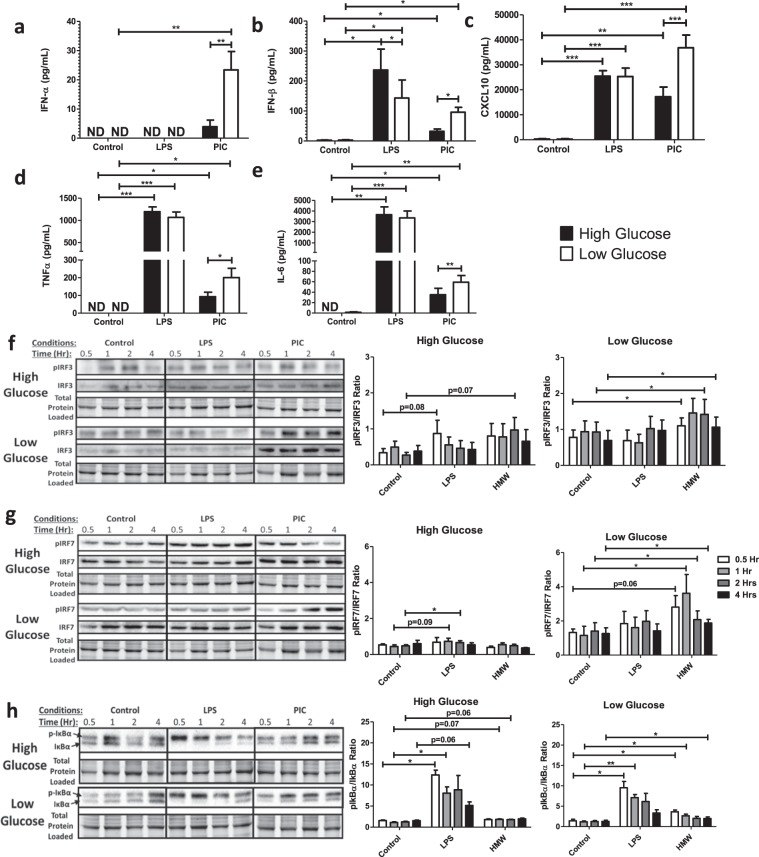
Low glucose conditions are associated with increased IRF activation and increased type I IFN production. Macrophages were stimulated with either 100 ng/mL LPS or 10 ng/mL PIC for 18 hours under high glucose (25 mM) or low glucose (0.5 mM) media conditions. Supernatant was collected for assessing antiviral (IFN-α, IFN-β, CXCL10) (**a**–**c**) and pro-inflammatory (TNF-α, IL-6) cytokine (**d**,**e**) expression. Cell lysates were harvested to quantify p-IRF3 and total IRF3 (**f**), p-IRF7 and total IRF7 (**g**) and p-Iκbα and total Iκbα (**h**) expression *via* immunoblotting. Data represents mean ± SEM of four individual mice (*p < 0.05, **p < 0.01, and ***p < 0.001). For visualization purposes, the western blot images were cropped, but full-length blots and gel images can be found in Supplemental Fig. S5.

Macrophages are highly plastic cells whose responses are modified by environmental cues including nutrient availability^38–41^. A recent study in BMM showed that macrophages are less dependent on OXPHOS activity under condition of high glucose (>10 mM) and preferentially use aerobic glycolysis to rapidly produce ATP^23^. Given the differential ability of BMM to reprogram OXPHOS activity based on glucose availability, we next evaluated how glucose levels affected TLR3 and TLR4 signaling and cytokine production. Most studies have been performed in standard culture conditions, which represent supra-physiological concentrations of glucose. For these studies, BMM were stimulated with LPS (100 ng/ml) and PIC (10 ng/ml) in standard (25 mM) and low glucose (0.5 mM) conditions. For the remainder of this paper, standard culture conditions will be referred as high glucose. Glucose levels had no effect on pro-inflammatory cytokine production in LPS stimulated cells (Fig. 4) suggesting that even low glucose levels are sufficient to support TLR4 responses. Conversely, low glucose conditions increased pro-inflammatory (TNF-α, IL-6) and IFN-associated cytokine production (IFN-α, IFN-β, CXCL10) following PIC stimulation suggesting that glucose availability may fine tune the magnitude of the TLR3 response. Interestingly, we found that high levels of glucose increase baseline OCR levels, spare respiratory capacity and ATP production in untreated/resting cells. However, following PIC stimulation, high glucose further reduced basal OCR, spare respiratory capacity and ATP production suggesting these conditions may alter TLR3 associated mitochondrial reprogramming (Supplementary Fig. S1).

To determine if this amplification was associated with altered signaling, we next evaluated alterations in TLR3 and TLR4 adaptor protein expression and transcription factor phosphorylation following stimulation with LPS and PIC under high and low glucose conditions. While TRIF and TRAF6 expression was not affected by glucose levels (Supplementary Fig. S2), low glucose was associated with a dramatic increase in phosphorylated IRF3 relative to total IRF3 in PIC-stimulated BMM after 0.5, 2 and 4 hours (Fig. 4f). Consistent with its more downstream role in TLR3 signaling^42,43^, we also found IRF7 phosphorylation was significantly elevated in low glucose conditions during the first four hours of activation (Fig. 4g). Furthermore, we found that levels of phosphorylated IκBα were increased in low glucose conditions in PIC but not LPS-stimulated cells, which may explain the increased TNF-α and IL-6 production following TLR3 engagement (Fig. 4h). Collectively, these results suggest that high glucose may limit mitochondrial reprogramming and associated antiviral and pro-inflammatory signaling and cytokine production in a TLR3 specific manner.

### Reduced glucose availability is associated with altered MMP and ETC complex expression following TLR engagement

**Figure 5:**
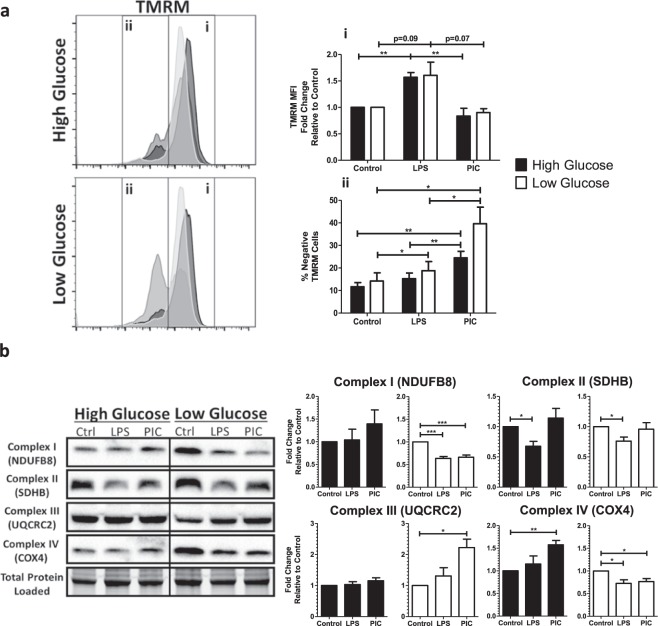
Poly(I:C) activation is linked to altered mitochondrial activity under low glucose conditions. BMMs treated with LPS or PIC for 18 hours under high glucose or low glucose media conditions were characterized for differences in mitochondrial function. Tetramethylrhodamine (TMRM) staining was used to measure, *via* flow cytometry, mitochondrial membrane potential. (**a**) Core protein levels of Complexes I-IV of the electron transport chain was quantified *via* immunoblotting. **(b**) Data represents mean ± SEM of four individual mice (*p < 0.05, **p < 0.01, and ***p < 0.001). For visualization purposes, the western blot images were cropped, but full-length blots and gel images can be found in Supplemental Fig. S6.

To better understand how glucose levels affect TLR3 associated alterations in OXPHOS activity, we examined alterations in MMP and ETC complex expression in high and low glucose conditions. MMP was assessed using the fluorescent dye Tetramethylrhodamine (TMRM). As previously described^15^, LPS stimulation was associated with increased sequestration of TMRM by activated mitochondria (TMRM Mean Fluorescence Intensity [MFI]) in both high and low glucose conditions (Fig. 5a). Alternatively, PIC stimulation did not significantly increase levels of TMRM sequestration in positive cells. Instead, PIC was associated with a significant increase in the number of cells expressing low levels of TMRM, which further increased under low glucose conditions (24% vs. 38%, p = 0.09). To evaluate if altered ETC flux contributes to altered membrane potential, we examined ETC complex expression following LPS and PIC stimulation in high versus low glucose conditions. In high glucose conditions, alterations in expression were highly variable across animals. LPS stimulation moderately decreased complex II (SDHB) expression whereas PIC increased complex IV (COX4) (Fig. 5b). Alterations in ETC complex expression were more pronounced in low glucose conditions. Specifically, both LPS and PIC were associated with decreased expression of complexes I and IV. The only alteration unique to PIC in the low glucose condition was the significant increase in complex III (Fig. 5b). In addition to its role as a proton pump, complex III is a major generator of mtROS^44^ and may contribute to the amplification of inflammatory and antiviral cytokine production following TLR3 engagement.

### Flux through the ETC chain is required for inflammatory and antiviral cytokine production following TLR engagement

**Figure 6:**
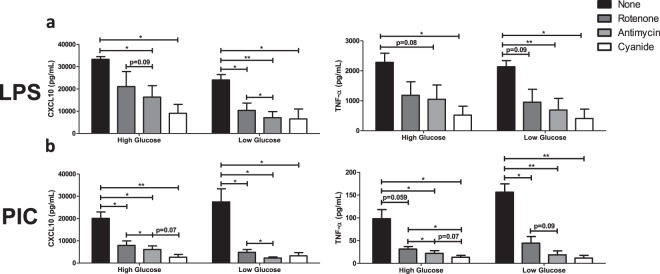
Targeting ETC activity reduces type I IFN-mediated responses during Poly(I:C) activation. LPS- (**a**) or PIC- (**b**) stimulated BMMs were co-treated with a panel of ETC inhibitors (Rotenone, Antimycin, Cyanide) to assess the importance of mitochondrial function for antiviral responses. CXCL10 and TNF-α cytokine secretion was measured after 18 hours in high glucose or low glucose media conditions. Data represents mean ± SEM of three individual mice (*p < 0.05, **p < 0.01, and ***p < 0.001).

To evaluate the relative contribution of specific ETC complexes on cytokine production, BMM were stimulated with LPS (100 ng/ml) and PIC (10 ng/ml) under high and low glucose conditions in the presence or absence of Rotenone (Complex I inhibitor), Antimycin (Complex III inhibitor) and Cyanide (Complex IV inhibitor). Inhibition of Complex III and IV significantly reduced CXCL10 (III: ↓51%; IV: ↓72%) and TNF production (III: ↓54%; IV: ↓77%) following LPS stimulation (Fig. 6a). Under low glucose conditions, inhibition of Complex I also limited LPS-associated cytokine production (CXCL10: ↓57%; TNF: ↓55%). Similarly, inhibition of Complexes I, III and IV significantly reduced inflammatory (TNF) and antiviral cytokine production (IFN-α, IFN-β, CXCL10) following PIC stimulation (Fig. 6b, Supplementary Fig. S3). The magnitude of this inhibition was further amplified under low glucose conditions, particularly for complex III (CXCL10: ↓70% vs. ↓92%; TNF-α: ↓78% vs. ↓89%; IFN-β: ↓79% vs. ↓88%). These results suggest that ETC flux is required for inflammatory and antiviral cytokine production and that under low glucose conditions, alterations in complex III expression may play a central role in the amplification of these responses following TLR3 engagement.

### Mitochondrial and cytosolic ROS accumulate in PIC stimulated BMM under low glucose conditions

**Figure 7:**
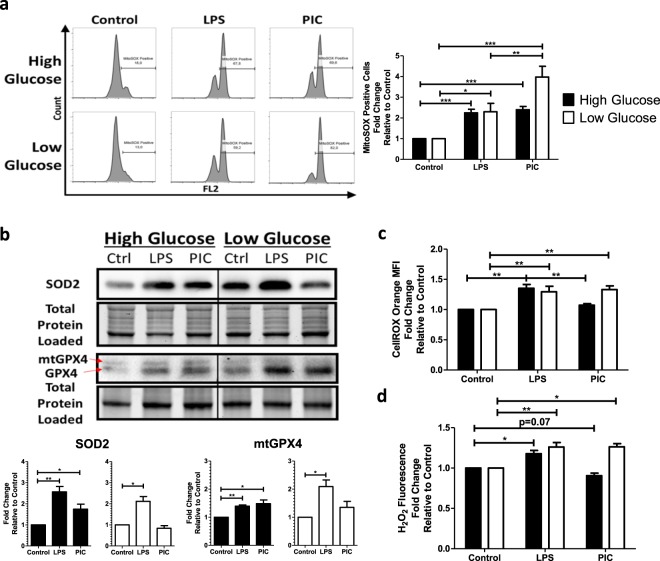
Poly(I:C) activation promotes mitochondrial ROS production and accumulation. Macrophages treated either with LPS or PIC for 18 hours under high glucose or low glucose media conditions were examined for differences in redox metabolism. Mitochondrial superoxide production was measured using MitoSOX Red^TM^ (**a**). Protein levels of antioxidant proteins superoxide dismutase (SOD2) and mitochondrial glutathione peroxidase 4 (mtGPX4) were measured via immunoblotting **(b)**. Cytosolic ROS production was measured using CellROX Orange **(c)**. Hydrogen peroxide levels were quantified using the Cell-based Hydrogen Peroxide Assay kit **(d)**. Data represents mean ± SEM of four individual mice (*p < 0.05, **p < 0.01, and ***p < 0.001). For visualization purposes, the western blot images were cropped, but full-length blots and gel images can be found in Supplemental Fig. S7.

Given the central role of complexes I and III in driving mitochondrial ROS production, we next quantified mitochondrial superoxide production using the fluorescent probe MitoSOX^TM^ Red. As previously reported^45^, LPS stimulation was associated with increased mitochondrial superoxide production compared to untreated BMM. This increase was unaffected by glucose availability (Fig. 7a). PIC stimulation was also associated with increased mitochondrial superoxide production (Fig. 7a); however, its production was further increased under low glucose conditions. To determine if this superoxide accumulation was associated with altered antioxidant expression, cellular levels of superoxide dismutase 2 (SOD2) and mitochondrial glutathione peroxidase 4 (mtGPX4) were evaluated *via* western blots. In high glucose conditions, both LPS and PIC stimulated cells significantly increased SOD2 (LPS-FC = 2.56 ± 0.44; PIC-FC = 1.75 ± 0.40) and mtGPX4 expression (LPS-FC = 1.40 ± 0.07; PIC-FC = 1.48 ± 0.22) levels (Fig. 7b). Alternatively, while LPS upregulated both antioxidant proteins under low glucose conditions (SOD2-FC = 2.11 ± 0.40; mtGPX4-FC = 2.09 ± 0.41), levels of SOD2 (FC = 0.83 ± 0.22) and mtGPX4 (FC = 1.35 ± 0.38) were not altered following PIC. This may contribute to the accumulation of superoxide in the mitochondria. To evaluate if these alterations affect the accumulation of cytosolic ROS, we used CellROX^TM^ Orange and quantified H_2_O_2_ levels in cell lysates. CellROX^TM^ Orange has a high affinity for hydroxyl radicals, H_2_O_2_ and superoxide. BMM treated with LPS exhibit increased levels of cytosolic ROS in both high and low glucose conditions (Fig. 7c). Conversely, increased cytosolic ROS was only observed in low glucose conditions following PIC stimulation (Fig. 7c,d). These results suggest that low glucose conditions are associated with increased mitochondrial and cytosolic ROS accumulation following PIC stimulation, which may contribute to the amplification of the cytokine response.

### Mitochondrial and cellular ROS play a central role in TLR3 and TLR4 associated cytokine production

**Figure 8:**
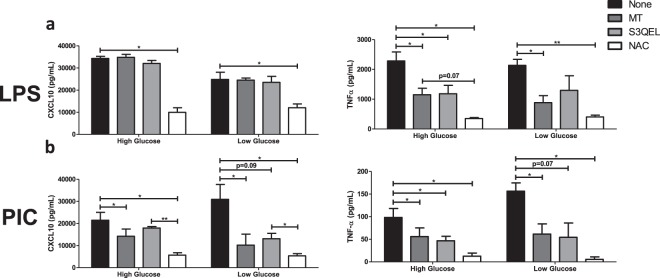
Type I IFN production can be inhibited by altering mtROS generation during Poly(I:C) activation. LPS (**a**) or PIC (**b**) stimulated BMMs were co-treated with a panel of mtROS (MT, S3QEL, NAC) modulators to assess the importance of mitochondrial function for antiviral responses. CXCL10 and TNF-α cytokine secretion was measured after 18 hours in high glucose or low glucose media conditions. Data represents mean ± SEM of three individual mice (*p < 0.05, **p < 0.01, and ***p < 0.001).

To evaluate if ROS contributes to cytokine production following TLR3 and TLR4 engagement, cells were stimulated with either LPS (100 ng/ml) or PIC (10 ng/ml) under high and low glucose conditions in the presence or absence of antioxidants (MitoTEMPO, *N*-acetylcysteine) or an inhibitor of superoxide production (S3QEL). MitoTEMPO (MT) is a mitochondria-specific antioxidant that selectively scavenges mitochondrial superoxide. Alternatively, *N*-acetylcysteine (NAC) boosts glutathione synthesis reducing total overall cellular ROS production. S3QEL selectively inhibits superoxide production from the outer Q-binding site of the ETC complex III without altering OXPHOS. Inhibition of mitochondrial ROS had differential effects on cytokine production following LPS stimulation (Fig. 8a; Supplementary Fig. S4). Whereas TNF-α (MT: ↓50%; S3QEL: ↓48%) and IFN-β levels (MT: ↓60%; S3QEL: ↓61%) were decreased following MT and S3QEL production, CXCL10 levels were unaffected. CXCL10 production was only reduced following NAC treatment (CXCL10: ↓71%) suggesting cytosolic ROS may play a more important role in its production following LPS stimulation. Alternatively, inhibition of mitochondrial ROS significantly reduced TNF-α, CXCL10, IFN-α and IFN-β production in PIC-activated macrophages (Fig. 8b; Supplementary Fig. S4). The magnitude of S3QEL inhibition was further amplified under low glucose conditions (TNF-α: ↓54% vs. ↓66%; CXCL10: not significant vs. ↓66%, IFN-β: ↓61% vs. ↓73%) suggesting complex III plays an important role in the amplification of the TLR3 responses. Similar to LPS, NAC treatment had the most pronounced effects on cytokine production in both and high low glucose conditions suggesting that both mitochondrial and cytosolic ROS contribute to TLR3 mediate cytokine production.

## Discussion

Increasing evidence suggests that mitochondria play a critical role in driving innate immune responses against bacteria and viruses^15,16,20–23,33^. However, it is unclear if specific features of mitochondrial reprogramming contribute to pathogen specific immune responses or how nutrient availability may affect these processes. In the current study, we found that TLR3 and TLR4 engagement uniquely remodeled ETC complex expression, resulting in differential accumulation of mitochondrial and cytosolic ROS. This differential ROS production is required to support ligand specific inflammatory and antiviral cytokine profiles. We also found that the magnitude of TLR3 but not TLR4 responses were modulated by glucose availability. Under conditions of low glucose, TLR3 engagement was associated with increased ETC complex III expression, increased mitochondrial and cytosolic ROS and increased inflammatory and antiviral cytokine production. This increased cytokine production was selectively reversed by targeting superoxide production from the outer Q-binding site of the ETC complex III. Collectively, these findings suggest that the ETC may act as a selective rheostat of macrophage function that regulates not only the nature (antibacterial vs. antiviral) but the magnitude of the response, which may depend on nutrient availability.

It is widely accepted that inflammatory macrophages undergo metabolic reprogramming to support cytokine production and effector functions. In LPS-stimulated cells, reprogramming is associated with a near complete inhibition of OXPHOS and an increased reliance on aerobic glycolysis to support rapid energy production^9,15,17,36,46^. This switch is driven by altered flux through the tricarboxylic acid (TCA), which repurposes mitochondrial function to support superoxide production and drive intracellular anti-bacterial responses^15,17^. While it was initially assumed all “inflammatory” stimuli induce similar responses, increasing evidence suggests this may not be the case^22,23,47^. In the current study, we found that PIC stimulation inhibited OXPHOS activity in a dose dependant manner. However, even at its highest concentrations (10 μg/ml), PIC did not completely inhibit OXPHOS activity and some level of cellular respiration was maintained. Consistent with these findings, Yoshizumi *et al*. found that RLR-mediated responses in macrophages are dependent on OXPHOS both *in vitro* and *in vivo*. In BMM, disruption of cellular respiration severely impaired RLR induced interferon and proinflammatory cytokine production^23^. In mice, inhibition of OXPHOS was found to increase susceptibility to viral infection and induce significant inflammation in the lung^23^. Wu *et al*. found that TLR9 engagement and type I IFN production in plasmacytoid dendritic cells was associated with increased OXPHOS activity. This increase was fueled by fatty acid oxidation (FAO) and was required for full cellular activation^48^. *In vivo*, inhibition of FAO resulted in a diminished capacity to control lymphocytic choriomeningitis virus^48^. Several IFN-stimulated genes, such as ISG15, have also been linked to the regulation of mitochondrial function during viral infection suggesting a secondary wave of mitochondrial reprogramming may occur following the TLR engagement and the induction of type I IFN responses^49^. Collectively, these studies suggest that some level of OXPHOS activity may be required to mount functional antiviral immune responses but that these responses may vary by ligand and cell type.

In addition to altered cellular respiration, LPS and PIC stimulation were associated with alterations in MMP. MMP is generated by the proton pumps of the ETC (Complexes I, III and IV) to support mitochondrial ATP production^50^. Various studies have reported altered MMP following macrophage activation. Mills *et al*. found that LPS stimulation was associated with increased MMP *via* reverse electron flow (RET). This RET was required to drive electrons back towards Complex I in order to support mitochondrial ROS production and antimicrobial effector functions^15^. Koshiba *et al*., have found MMP is required for MAVS-mediated antiviral signaling. Specifically, they found that inhibition of mitochondrial fusion resulted in a widespread loss of MMP. This loss in MMP correlated with the level of inhibition of RLR-induced antiviral responses^22^. Unlike LPS, we found that PIC stimulation was associated with decreased MMP in a subset of cells. While it is unclear what exactly these cells represent, Tal *et al*. reported that when autophagy is inhibited, increased accumulation of dysfunctional mitochondria results in increased mitochondrial ROS production, which drives excess RLR signaling^26^. Further studies are required to determine if this subset of TMRM low cells are the main producers of ROS in our model system.

Recent studies have provided evidence that changes in the ETC, particularly in complex I and II, contribute to the regulation of antibacterial immune responses^15,33^ however, it is unclear if similar remodeling occurs during antiviral responses. Here, we found that PIC stimulated cells undergo differential remodeling of the ETC, particularly with limited glucose availability. Standard cell culture conditions represent supra-physiological levels of glucose (25 mM vs. 5–7 mM in fasting blood from non-diabetic individuals^51^) and may alter mitochondrial reprogramming *in vitro*^52^. Under low glucose conditions, both LPS and PIC were found to downregulate complex I and IV expression. Interestingly, only PIC stimulation also increased complex III expression. This increased expression was associated with the amplification of the TLR3 cytokine production, which was reversed by the selective inhibition of ROS production by complex III. In support of our results, others have linked complex III to immune activation and function. In T cells, Sena *et al*. demonstrated that specific deletion of Rieske iron-sulfur protein (RISP), an essential component of Complex III, reduced mtROS production, nuclear factor of activated T cells (NFAT) activation, IL-2-mediated T cell activation and antigen-specific expansion *in vivo*^53^. Alternatively, ablation of complex III in regulatory T cells has been shown to reduce their inhibitory capacity without altering cell proliferation and survival^54^. In macrophages, listeria infection has been shown to increase ROS production *via* complex III, which drives NF-kappa-B essential modulator (NEMO) dimerization, increasing inhibitor of nuclear factor kappa-B kinase (IKK) activation, NF-κB signaling and cytokine production^47^ Our study is among the first to identify associations between complex III mediated ROS production and TLR3 antiviral immune responses.

While ROS are generally considered toxic and damaging, increasing evidence suggests they also influence cellular signaling^55,56^. Superoxide and its more stable derivative hydrogen peroxide have been shown to regulate a variety of biological responses such as cell proliferation, differentiation, and migration^55^. In the current study, we found that both mitochondrial superoxide and cytosolic ROS contribute to inflammatory and antiviral cytokine production following TLR engagement and that differential accumulation of ROS across these compartments may contribute to pathogen specific responses. Furthermore, under low glucose conditions, we found PIC but not LPS was associated with increased mitochondrial superoxide and cytosolic ROS production, which consequentially amplified cytokine production in these cells. These results suggest that the dynamic regulation of ROS production, likely through the modulation of complex III, may act as a rheostat that regulates the magnitude of antiviral immune responses. Consistent with these findings, Agod *et al*. found that mitochondrial superoxide drives increased MAVS protein expression in plasmacytoid DCs, increasing Akt and IRF3 activation and subsequent type I IFN production^57^. Wang *et al*. showed shRNA knockout of SOD2 in cell lines increased viral replication and reduced antiviral responses^58^, likely a result of decreased mitochondrial H_2_O_2_ production, a known redox-sensitive activator of NF-κB and IRF signalling^47,59–61^. While we believe that mitochondrial superoxide and associated hydrogen peroxide production are the main drivers of cytokine production in our system, we cannot exclude the possibility that alternative cytosolic sources of ROS may also contribute. NADPH oxidase (NOX)-generated ROS during respiratory syncytial virus (RSV) and herpes simplex virus (HSV) infections can activate both NF-κB and IRF signalling^60,61^. Similarly, Yang *et al*. reported that high concentrations of PIC increase NOX2 activity and ROS production in BMMs, which was required for signal transduction and activator of transcription 1 (STAT1)-mediated signalling^62^. A similar phenomenon was observed by To *et al*. using TLR7 ligands^63^. Further studies are required to determine the specific contribution of mitochondrial vs. cytosolic derived ROS in driving these processes and the distinct roles of superoxide vs. hydrogen peroxide on signaling and effector function.

Taken together, our results suggest that dynamic remodeling of the ETC complex expression represents a mechanism by which macrophages modulate cytokine production following TLR engagement. We found that this remodeling was associated with differential accumulation of mitochondrial vs. cytosolic ROS, which may drive ligand specific cytokine profiles. We hypothesize that this differential accumulation may be driven by dependence on complex I (LPS) versus complex III (PIC) for ROS production. Specifically, that LPS associated ROS production depend on RET whereas PIC depends on the maintenance of low levels of OXPHOS activity. Interestingly, we also found that TLR3 but not TLR4 associated mitochondrial reprogramming was dependent on glucose availability in the microenvironment. Supra-physiological levels of glucose have been shown to decrease a cell’s dependence on mitochondria for energy production^52,64^. Similarly, our results suggest high glucose conditions may also alter mitochondrial reprogramming associated with TLR3 engagement. Accordingly, it is important to develop a detailed understanding of these processes in a variety of bacterial and viral infections to identify new therapeutic approaches to help boost specific and functional effector functions.

## Methods

### Reagents

A list of all reagents, chemicals or kits used in this study can be found in Table S1. Lipopolysaccharide (LPS) and high molecular weight Poly(I:C) (PIC) were purchased from InvivoGen. MitoTEMPO (MT), *N*-acetylcysteine (NAC), antimycin A (AA), rotenone (ROT), potassium cyanide, 2-deoxyglucose (2-DG), oligomycin (OM) and carbonyl cyanide-*p*-trifluoromethoxyphenylhydrazone (FCCP) were acquired from Sigma-Aldrich while S3QEL-2 was purchased from Cedarlane. IL-1β, IL-6, IL-10, TNF-α, and CXCL10 ELISA kits were purchased from R&D Systems. The IFN-α/IFN-β 2-Plex Mouse ProcartaPlex^TM^ Luminex Panel kit used was from Invitrogen. Tetramethylrhodamine, methyl ester (TMRM), MitoSox Red and CellROX Orange probes were from ThermoFisher. Antibodies against Complex II (SDHB) was from Abcam while antibodies recognizing SOD2 was purchased from Cell Signalling Technology. Antibodies targeting IRF3, pIRF3 (Ser385), IRF7, pIRF7 (Ser477), GPX4, Iκbα, Complexes I (NDUFB8), III (UQCRC2) and IV (COX4) were purchased from ThermoFisher.

### BMM culturing and stimulation

Total bone marrow cells were collected from the tibias and femurs of 6–13-week-old C57BL/6 mice, cryopreserved in a 90% FBS/10% DMSO solution, and frozen until use. All animal procedures were approved by the Carleton University Animal Care Committee and were conducted in accordance with the guidelines provided by the Canadian Council for Animal Care. Cells were cultured for ten days in DMEM media with 10% fetal bovine serum, 1% penicillin/streptomycin (Life Technologies), and 15% L929 fibroblast cell-conditioned medium on a 100 mm Petri dish as previously described^65^. On day 10, differentiated bone marrow-derived macrophages (BMM) were detached, counted and plated into tissue-culture treated plates at 1 × 10^6^ cells/mL. BMMs were stimulated with 100 ng/mL LPS, 10 ng/mL or 10 μg/mL PIC under high (DMEM medium supplemented with 25 mM glucose) or low glucose conditions (DMEM medium supplemented with 0.5 mM glucose). The relative contribution of the ETC and ROS production on BMM inflammatory and antiviral cytokine production were assessed by co-treating stimulated cells with 1 μM ROT, 5 μM AA, 5 mM cyanide, 500 μM MT, 5 mM NAC, or 5 μM S3QEL-2.

### Cytokine quantification

After the 18-hour stimulation, cytokine production was assessed in culture supernatants. IL-1β, IL-6, IL-10, TNF-α, and CXCL10 levels were assessed by ELISAs according to the manufacture's instructions (R&D Systems). IFN-α and IFN-β levels were measured using IFN-α/IFN-β 2-Plex Mouse ProcartaPlex^TM^ Luminex Panel (Invitrogen).

### Western blot analysis

Untreated and stimulated BMMs (1 × 10^6^ cells) were lysed directly in the cell culture vessel using Pierce RIPA buffer (ThermoFisher) supplemented with HALT^TM^ Protease and Phosphatase Inhibitor (ThermoFisher). Total protein was quantified using the DC assay (Bio-Rad) and resolved on a TGX^TM^ FastCast^TM^ Acrylamide gels (Bio-Rad). Gels were imaged directly using the Stain-Free application of a ChemiDoc XR (Bio-Rad) prior to transferring onto a PVDF membrane. Membranes were blocked overnight in 5% non-fat dry milk (w/v), washed and incubated overnight with the appropriate primary antibody. Horseradish peroxidase-conjugated secondary antibodies and Clarity^TM^ Western ECL Blotting Substrate (Bio-Rad) were used to visualize specified protein bands. Protein densitometry was analyzed according to previously described methodology^66^. In brief, band intensity of target proteins was normalized relative to the total protein levels in each respective lane using the Bio-Rad Stain-Free application. Expression of most target proteins were normalized relative to the control sample and presented as a fold change value. Alterations in phosphorylated protein expression were calculated as the increase in phosphorylation as proportion of the total protein. Levels were then compared between the control vs. the treated groups (LPS and PIC) under low and high glucose conditions.

### Assessment of mitochondrial function by flow cytometry

BMMs were plated on 100 mm Petri dishes and stimulated with 100 ng/mL LPS or 10 ng/mL and 10 μg/mL PIC for 18 hours. Cells were then washed and stained with fluorescent probes according to the manufacturer’s instructions (30 mins treatment at 37 °C in select solutions). Mitochondrial membrane potential was measured using 10 nM TMRM. Mitochondrial and Cellular ROS were monitored using 2.5 μM MitoSOX Red in PBS and 5 μM CellROX Orange in both high and low glucose conditions, respectively. Cellular levels of fluorescence were quantified using an Attune NxT Flow Cytometer (ThermoFisher) and the results were analyzed using FlowJo Software. Results are reported as the percentage of positive cells and as mean fluorescence intensity (MFI), the latter being used to describe the level of expression on a population of positive cells.

### Quantification of cellular hydrogen peroxide production

BMMs were plated onto 96-well black plates at 50,000 cells/well and stimulated with 100 ng/mL LPS or 10 ng/mL PIC under high or low glucose conditions for 1 hour. Cells were then washed before using the Cell-based Hydrogen Peroxide Assay Kit (Abcam) to measure H_2_O_2_ production. Cells were incubated with the AbGreen H_2_O_2_ indicator for 30 minutes before monitoring the relative difference in fluorescence using a fluorescence microplate reader (490 nm Ex/520 nm Em).

### Metabolic extracellular flux analysis

BMMs were plated onto Seahorse XFp cell culture miniplates at 50,000 cells/well (Seahorse Bioscience) and stimulated with 100 ng/mL LPS or 10 ng/mL and 10 μg/mL PIC for 18 hours. Extracellular acidification rate (ECAR) and oxygen consumption rate (OCR) were evaluated using a XFp Flux Analyzer (Seahorse Bioscience). Baseline ECAR and changes in glycolytic rate were assessed using the Seahorse XFp Glycolytic Rate Assay Kit (Agilent) according to the manufacturer’s instructions. Basal respiration, ATP production-coupled respiration, maximal and reserve capacities and non-mitochondrial respiration were assessed using the Seahorse XFp Cell Mito Stress Test Kit (Agilent).

### Statistical analyses

Data used in this study was analyzed using GraphPad Prism software. Values shown represent the mean ± SEM of biological replicates, where the number of replicates are reported in the figure legends. Statistical significance was calculated using a paired Student’s *t*-test (*p < 0.05, **p < 0.01, and ***p < 0.001).

## Supplementary information

Supplemental Information
