# Supplementary material for: Differential remodeling of the electron transport chain is required to support TLR3 and TLR4 signaling and cytokine production in macrophages
Source: Sci Rep. 2019 Dec 11;9:18801. doi: 10.1038/s41598-019-55295-4 (PMC6906364; doi:10.1038/s41598-019-55295-4)

Differential remodeling of the electron transport chain is required to support TLR3 and TLR4 signaling and cytokine production in macrophages

Duale Ahmed[1], David Roy[2], Allison Jaworski[2], Alex Edwards[3], Alfonso Abizaid[3], Ashok Kumar[4,5], Ashkan Golshani[1], Edana Cassol[2]*

[1]Department of Biology, Carleton University, Ottawa, Ontario, Canada
[2]Department of Health Sciences, Carleton University, Ottawa, Ontario, Canada
[3]Department of Neuroscience, Carleton University, Ottawa, Ontario, Canada
[4]Department of Pathology and Laboratory Medicine, Faculty of Medicine, University of Ottawa,
[5]Department of Pathology, The Children's Hospital of Eastern Ontario Research Institute, Ottawa, ON K1H 8L1

Supplementary Information:

Supplementary Table S1: List of reagents, chemical or kit used in this study, referenced from Methods section.

Supplementary Figure S1: PIC activation is associated with increased OXPHOS function under low glucose conditions. BMDMs were plated onto Seahorse XFp miniplates and treated with 10μg/mL PIC for 18 hours. OXPHOS function was assessed *via* successive Oligomycin (Oligo), Carbonyl cyanide-*p*-trifluoromethoxyphenylhydrazone (FCCP), and Rot/AA injections **(a)**. Quantification of the spare respiratory capacity percentage (SRC%) **(b)** and ATP production **(c)**. Data shown represents a test run using one representative animal.

Supplementary Figure S2: TRIF and TRAF6 expression during PIC activation is not affected by glucose levels. Macrophages stimulated either with LPS or PIC for 18 hours under high glucose **(a)** or low glucose **(b)** media conditions were examined for differences in TLR signaling. Protein levels of TRIF and TRAF6 were measured via immunoblotting. Data shown represents blots from a representative animal while data shown represents mean ± SEM of two individual mice.

Supplementary Figure S3: Targeting ETC function leads to complete loss of IFN responses. LPS- **(a)** or PIC- **(b)** stimulated BMDMs were co-treated with a panel of ETC (Rotenone, Antimycin, Cyanide) inhibitors to assess the importance of mitochondrial function to antiviral responses. CXCL10 and TNF-α cytokine secretion was measured after 18 hours in high glucose or low glucose media conditions. Data shown represents a mean of two individual animals.

Supplementary Figure S4: Targeting mitochondrial ROS leads to loss of type I IFN production. LPS- **(a)** or PIC- **(b)** stimulated BMDMs were co-treated with a panel of mtROS (MT, S3QEL, NAC) modulators to assess the importance of mitochondrial function to antiviral responses. CXCL10 and TNF-α cytokine secretion was measured after 18 hours in high glucose or low glucose media conditions. Data shown represents a mean of two individual animals.

Supplementary Figure S6: Uncropped Images of Figure 4

Supplementary Figure S7: Uncropped Images of Figure 5

Supplementary Figure S8: Uncropped Images of Figure 7

Supplementary Figure S9: Uncropped Images of Supplementary Figure S2

Supplementary Table S1: List of reagents, chemical or kit used in this study.

Reagent or Resource				
**Antibodies**	**Supplier**	**Catalog Number**	**Additional Information**	
			**Isotype**	**Host**
SDHB Monoclonal Antibody	Abcam	ab14714	IgG2a	Mouse
SOD2 (D3X8F) XP® monoclonal antibody	Cell Signalling Technology	13141	IgG	Rabbit
IRF3 Polyclonal Antibody	ThermoFisher	PA5-20086	IgG	Rabbit
Phospho-IRF3 (Ser385) Polyclonal Antibody	ThermoFisher	PA5-38285	IgG	Rabbit
IRF7 Polyclonal Antibody	ThermoFisher	PA5-79519	IgG	Rabbit
Phospho-IRF7 (Ser477) Polyclonal Antibody	ThermoFisher	PA5-64834	IgG	Rabbit
NDUFB8 Monoclonal Antibody (Complex I)	ThermoFisher	459210	IgG1, kappa	Mouse
UQCRC2 Polyclonal Antibody (Complex III)	ThermoFisher	PA5-30204	IgG	Rabbit
COX4/Complex IV Monoclonal Antibody	ThermoFisher	A21348	IgG2a, kappa	Mouse
GPX4 Polyclonal Antibody	ThermoFisher	PA5-79321	IgG	Rabbit
Iκbα Polyclonal Antibody	ThermoFisher	PA5-22120	IgG	Rabbit
Anti-Mouse IgG HRP-linked Secondary Antibody	Abcam	ab6789	IgG	Goat
Anti-Rabbit IgG, HRP-linked Secondary Antibody	Cell Signalling Technology	7074	IgG	Goat
**Cell Culture**	**Supplier**	**Catalog Number**		
DMEM, high glucose, pyruvate	Life Technologies	11995-073		
DMEM, no glucose, no glutamine, no pyruvate	Life Technologies	A1443001		
FBS	Life Technologies	12484-028		
Penicillin-Streptomycin (10,000 U/mL)	Life Technologies	15140-122		
Glucose Solution (200g/L)	Life Technologies	A2494001		
Sodium Pyruvate (100 mM)	Life Technologies	11360070		
L-Glutamine (200 mM)	Life Technologies	25030081		
**Chemicals**	**Supplier**	**Catalog Number**		

Lipopolysaccharide	Invivogen	tlrl-eklps
Poly(I:C), High molecular weight	Invivogen	tlrl-pic-5
MitoTempo	Sigma	SML0737
N-Acetyl-L-cysteine	Sigma	A9165
Antimycin A	Sigma	A8674
Rotenone	Sigma	R8875
Oligomycin	Sigma	O4876
FCCP	Sigma	C2920
Potassium Cyanide	Sigma	60178
2-deoxyglucose	Sigma	D6134
S3QEL	Cedarlane	5735/10
**Commerical Kits**	**Supplier**	**Catalog Number**
Mouse IL-1 beta/IL-1F2 DuoSet ELISA Kit	R&D Systems	DY401
Mouse IL-6 DuoSet ELISA Kit	R&D Systems	DY406
Mouse IL-10 DuoSet ELISA Kit	R&D Systems	DY417
Mouse TNF-alpha DuoSet ELISA Kit	R&D Systems	DY410
Mouse CXCL10/IP-10/CRG-2 DuoSet ELISA Kit	R&D Systems	DY466
IFN alpha/IFN beta 2-Plex Mouse ProcartaPlex Panel	Life Technologies	EPX020-22187-901
Hydrogen Peroxide Assay Kit (Cell-based)	Abcam	ab138874
Seahorse XFp Glycolytic Rate Assay Kit	Aglient	103346-100
Seahorse XFp Cell Mito Stress Test Kit	Aglient	103010-100
**Reagents**	**Supplier**	**Catalog Number**
Image-iT™ TMRM Reagent	Invitrogen	I34361
MitoSOX™ Red Mitochondrial Superoxide Indicator	Invitrogen	M36008
CellROX™ Orange Reagent	Invitrogen	C10443
**Western Blot Accessories**	**Supplier**	**Catalog Number**
Halt™ Protease and Phosphatase Inhibitor Cocktail	ThermoFisher	78440

RIPA Lysis and Extraction Buffer	ThermoFisher	89900
DC™ Protein Assay Kit II	Bio-Rad	5000112
TGX Stain-Free™ FastCast™ Acrylamide Kit, 12%	Bio-Rad	1610185
Trans-Blot® Turbo™ RTA Mini PVDF Transfer Kit	Bio-Rad	1704272
Clarity™ Western ECL Substrate	Bio-Rad	1705060

Supplementary Figure S1

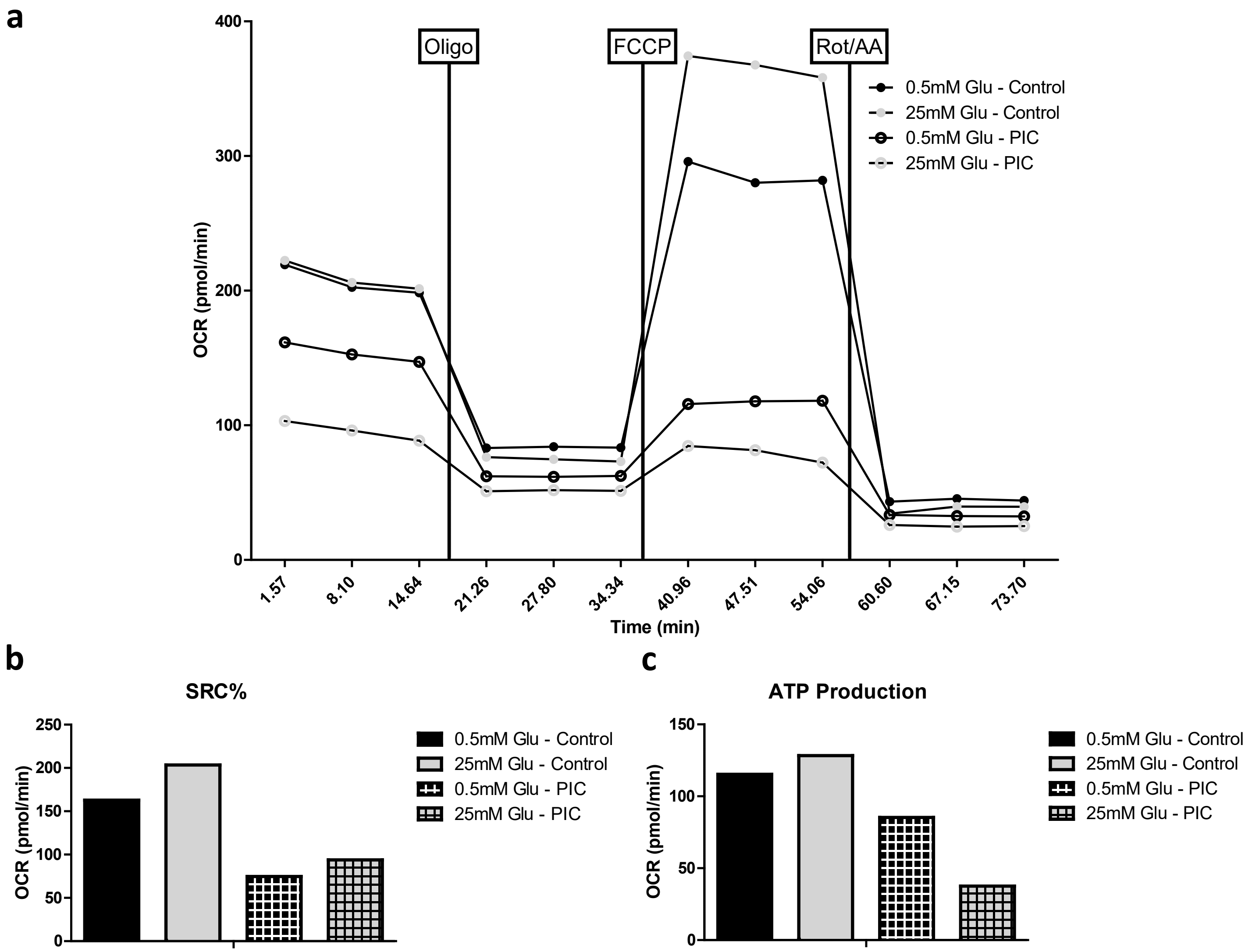

Supplementary Figure S2

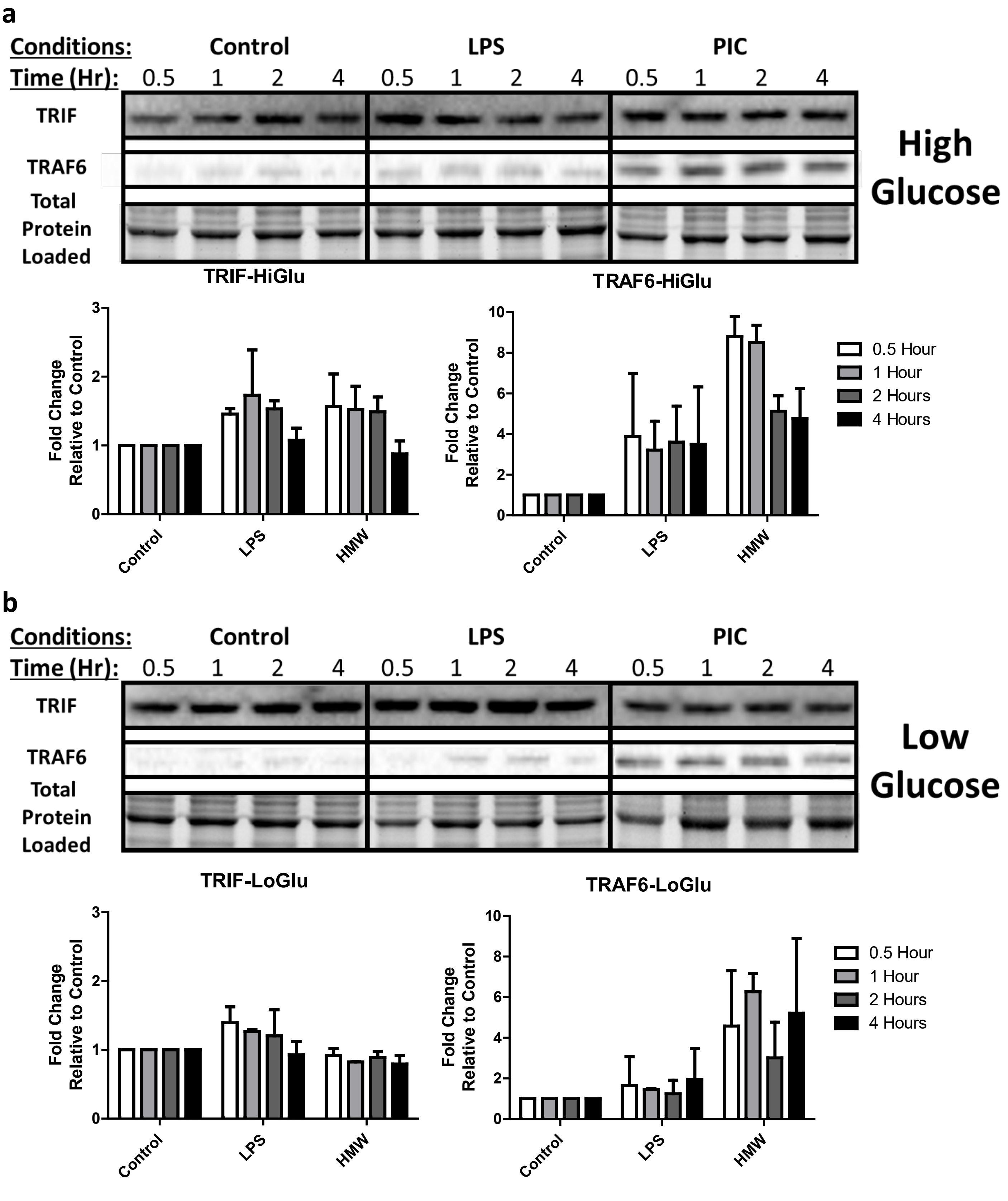

Supplementary Figure S3

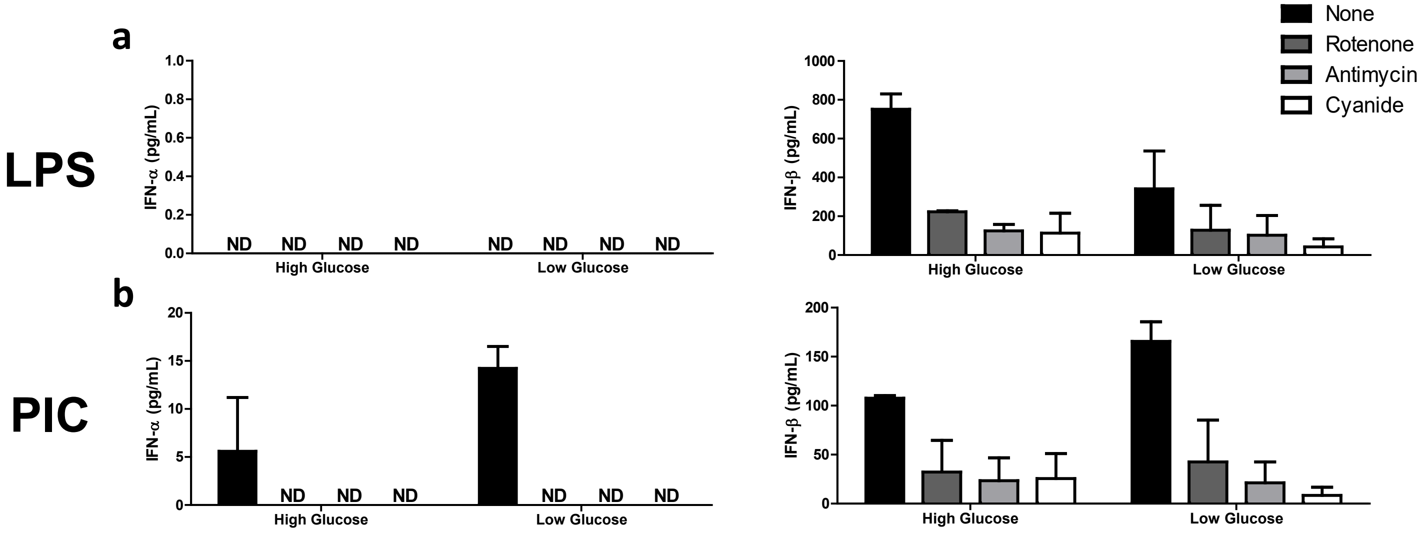

Supplementary Figure S4

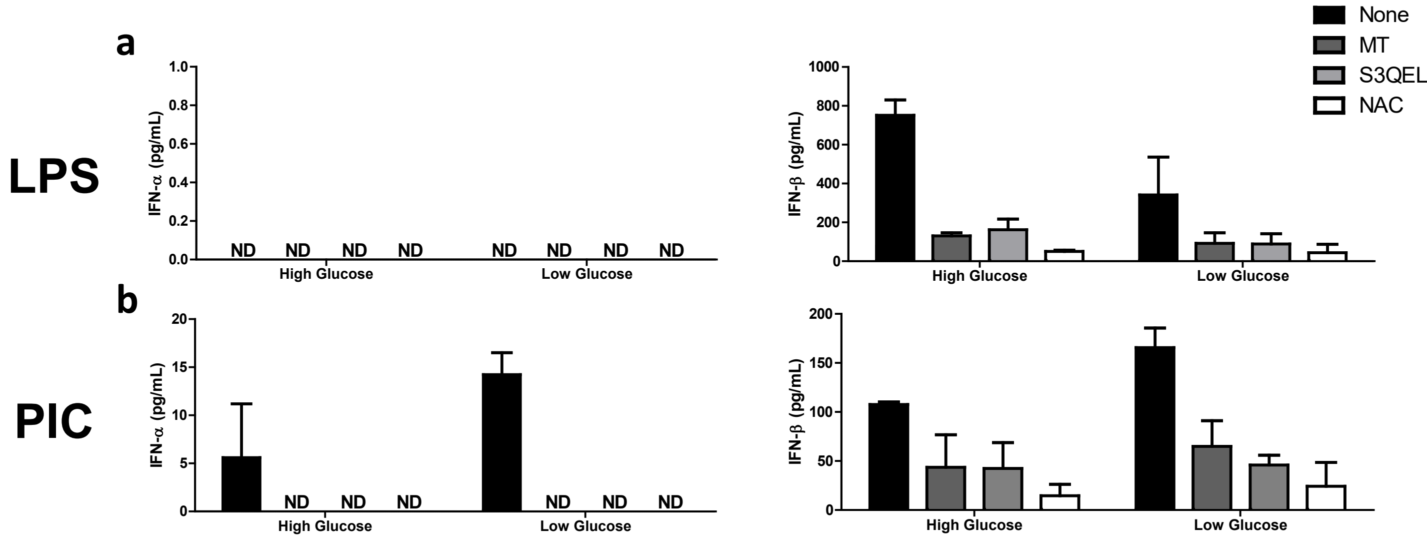

Supplementary Figure S5 – Uncropped Images of Figure 4

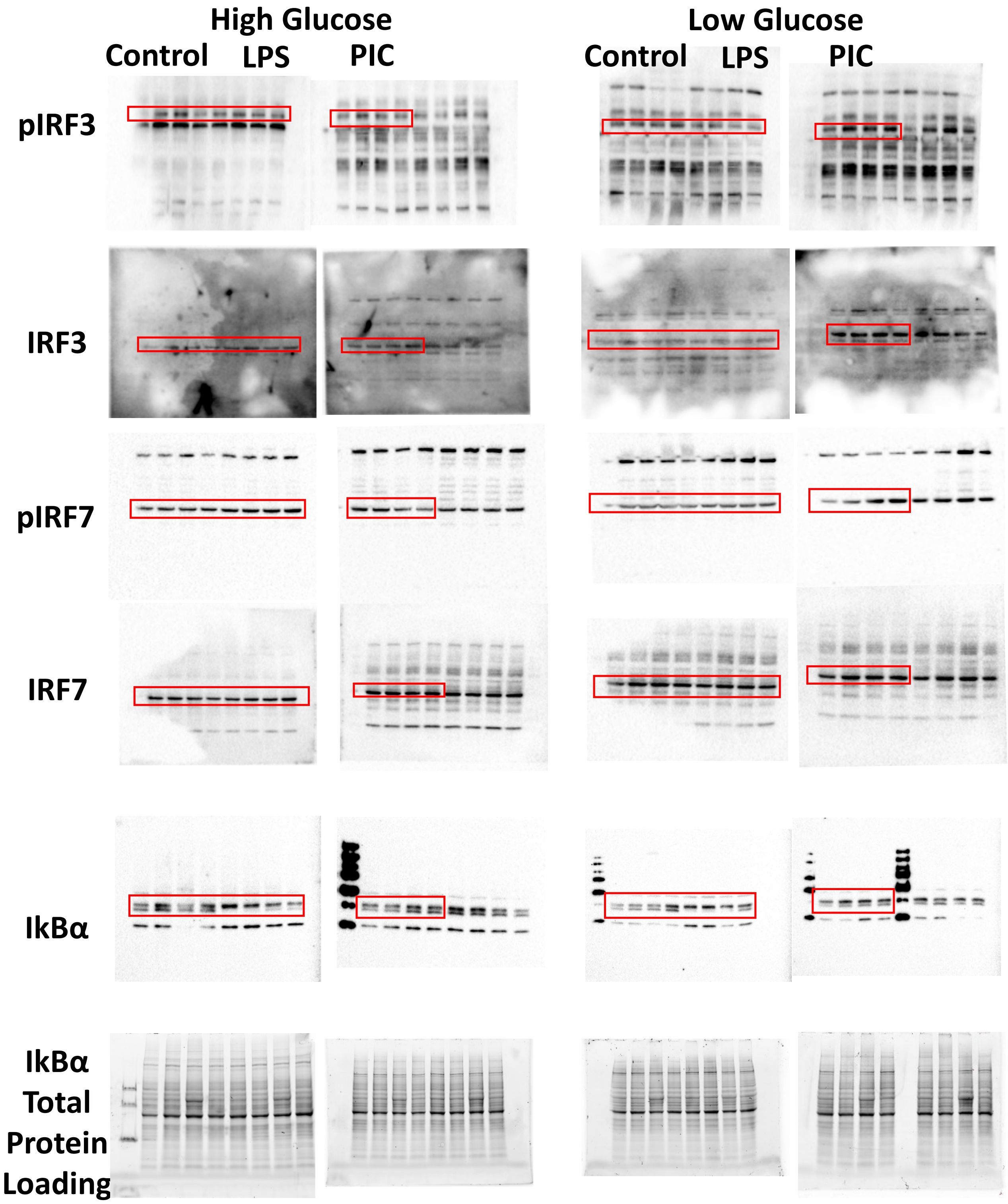

Supplementary Figure S6 – Uncropped Images of Figure 5

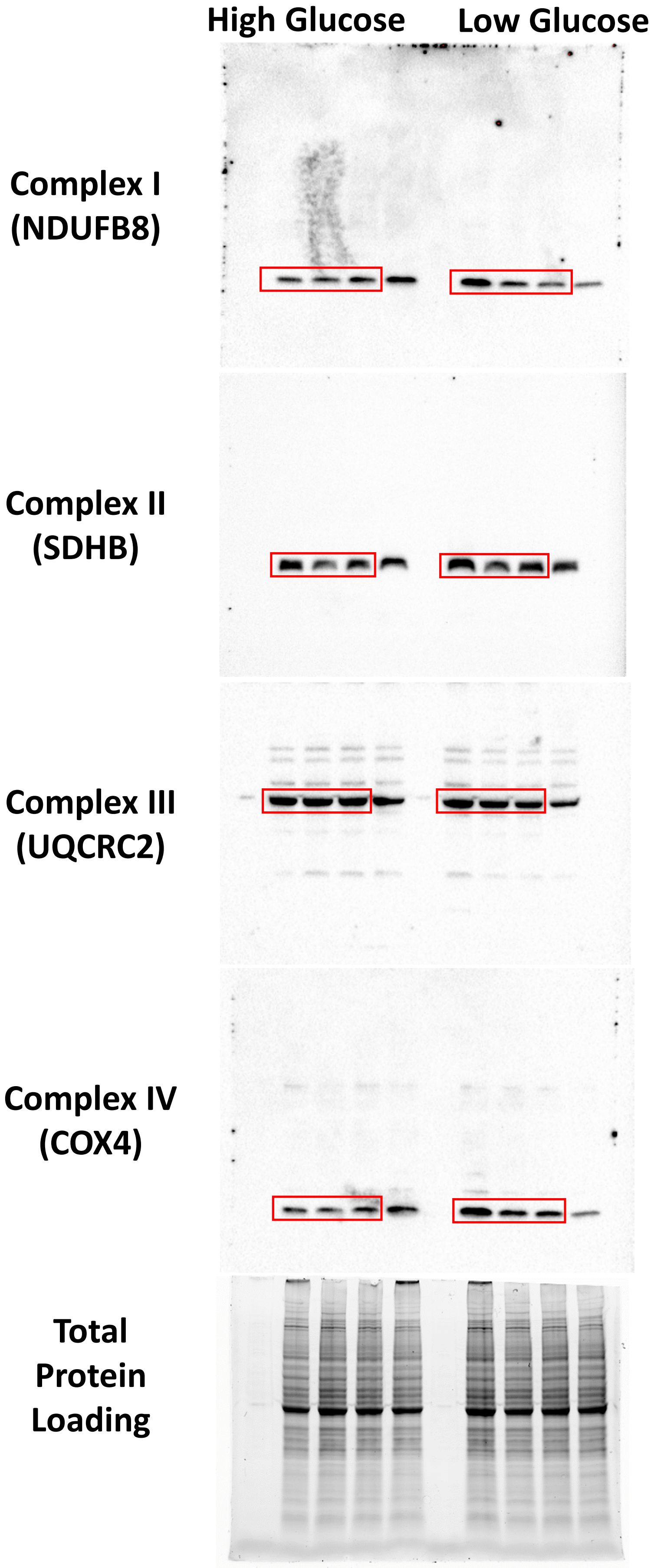

Supplementary Figure S7 – Uncropped Images of Figure 7

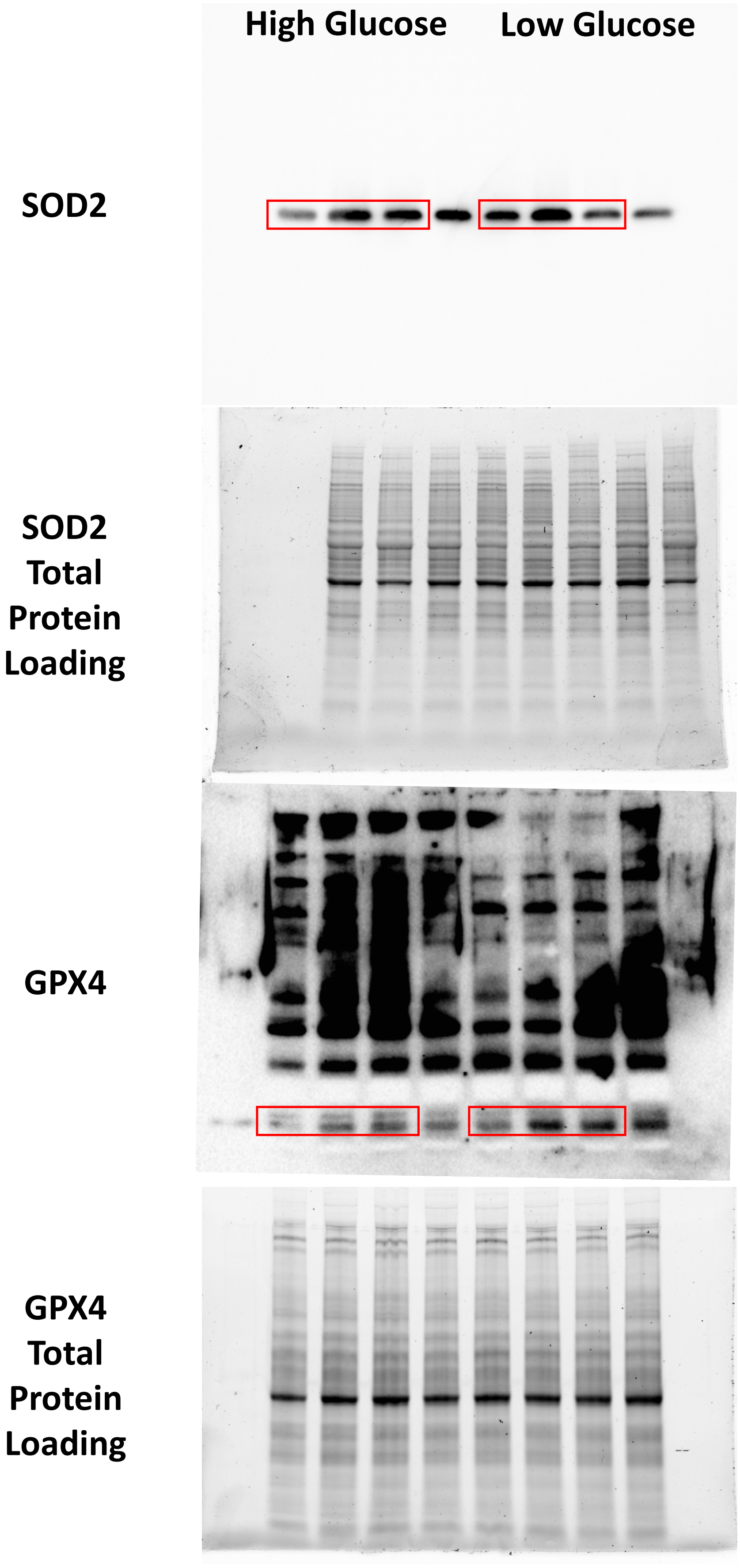

Supplementary Figure S8 – Uncropped Images of Supplementary Figure S2

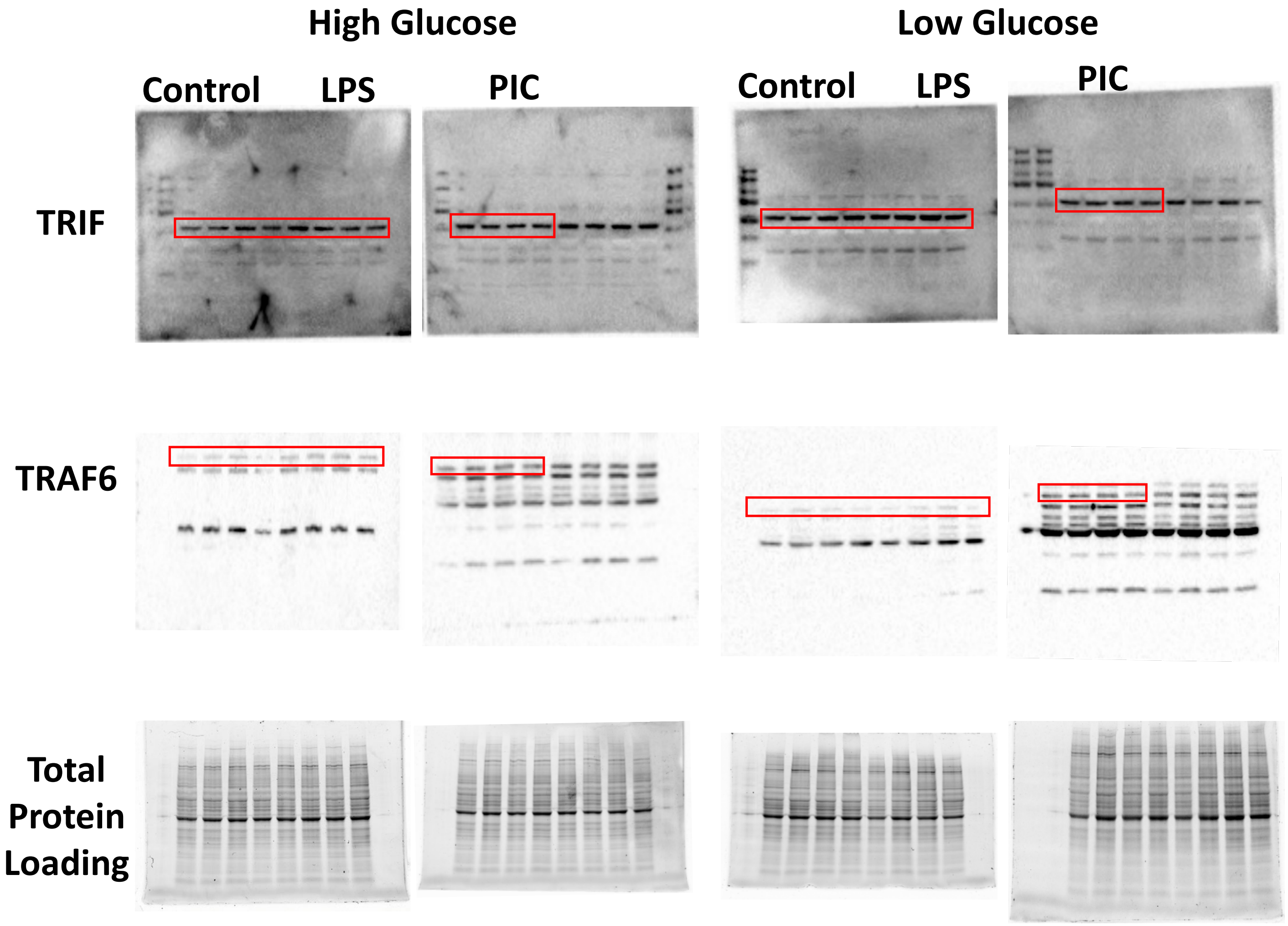

Supplement: Supplementary file 1 — Supplemental Information [file 41598_2019_55295_MOESM1_ESM.pdf]
